# Spatial distribution of the “*Mais Médicos* (More Doctors) Program” and social vulnerability: an analysis of the Brazilian metropolitan regions

**DOI:** 10.1186/s12960-020-00497-5

**Published:** 2020-08-05

**Authors:** Aimê Oliveira, Jorge Otávio Maia Barreto, Sidclei Queiroga de Araújo, Leonor Maria Pacheco Santos

**Affiliations:** 1 University of Brasília, UNB, Brasilia, Brazil; 2 Oswaldo Cruz Foundation, Fiocruz, Brasilia, Brazil

**Keywords:** Public health, Equity, Health vulnerability, More Doctors Program/*Mais Médicos* Program

## Abstract

**Background:**

The “*Mais Médicos* (More Doctors) Program” established in 2013 by the Brazilian Government aimed to reduce inequalities by means of an emergency provision of physicians, the improvement of medical care service in the Brazilian Unified Health System, and the expansion of medical education training in Brazil. In this context, equity should be considered when defining priorities and allocating resources. This study describes the distribution of physicians for the Program in five Brazilian metropolitan regions (MRs) and analyses whether the most vulnerable areas within each one of these regions had been prioritized in compliance with the legislation framework of the program.

**Methods:**

This is a quantitative cross-sectional study. Official secondary data was analyzed to verify the relationship between the Index of Social Vulnerability, set up by the Institute of Applied Economic Research, and the physician allocation provided by the Program. The data were organized into categories and quintiles. For spatialization purposes, the QGIS 3.4 Madeira software was used.

**Results:**

There are 2592 primary health care units, (in Portuguese, UBS), within the five MRs studied; 981 of these hosted at least one physician from the Program. In the Manaus, Recife, and the DF MRs, the 4th and 5th quintiles (the most vulnerable ones) hosted physicians in more significant proportions than the other quintiles, namely, 71.4%, 71.4%, and 52.2%, respectively, exceeding the national average (51.7%). It is worth mentioning that in the São Paulo MR, the units located in the most vulnerable quintiles (4th and 5th) also hosted physicians in proportions significantly higher than others (45.8%); however, this proportion did not reach 50%. There was no significant difference in the allocation of physicians in the Porto Alegre MR, indicating that there was no prioritization of the UBS according to vulnerability.

**Conclusions:**

These results appoint to the enormous gaps of vulnerability existing both between the analyzed MRs and internally in each one of them. It emphasizes the need for criteria for the allocation of physicians so as not to increase inequities. It also highlights the importance of the continuity of the “*Mais Médicos* (More Doctors) Program” in the metropolitan regions, above all, in areas of extreme vulnerabilities. On the other hand, they contribute to the national debate about the importance of public policies regarding constitutional rights related to access to health care and the relevance of primary care and the “*Mais Médicos* (More Doctors) Program” for the reduction of disparities regarding access to health care, especially for the citizens who live in regions of greater vulnerability, whether it is inside or outside large metropolitan regions.

## Background

Improvement in the population’s health and the reduction of inequalities are common goals for universal health systems. In order to reach these objectives, especially to achieve equity in the results of health care actions, it is mandatory that the health system is guided and grounded by the primary health care (PHC) focused on the social health determinants [1, 2], as health conditions are influenced by the socioeconomic, environmental, cultural, behavioral, and work conditions and the lifestyle, among other features, in addition to the access to quality health services [3].

PHC in Brazil is defined as “a set of individual, family, and collective health actions that involve the promotion, prevention, protection, diagnosis, treatment, rehabilitation, damage reduction, palliative care, and health surveillance” [4–6]. The Ministry of Health also states that the PHC must be based on the totality of actions to promote, diagnose, treat, and rehabilitate health, besides fostering the organization of the Brazilian Unified Health System (in Portuguese, SUS) [4, 6, 7]. It also emphasizes that its development must be carried out by means of interdisciplinary, democratic, and participative teamwork processes, using high-complexity and low-density technologies, characterized by the integrality of individual and collective care and the guarantee of bond and continuity (humanization) [8]. The collective dimension and the individual’s uniqueness must be its object of work whose purpose is based on solving the most frequent health issues and are more relevant [8, 9]. Thus, this level of care is paramount for the organization of the SUS. It is the gateway to foster the integrality of collective actions, without taking for granted the existing particularities [6].

The Brazilian socioeconomic inequality is seen throughout very heterogeneous territories and even coexists within the municipalities, especially in Brazilian capital cities and inside the large metropolitan regions. The unequal income distribution reflects such disparities in neighboring populations, and it contributes to the inefficiency in the access to health care and the distribution of resources, which directly impacts those individuals who live within a context of social vulnerability associated to poverty [10]. Social justice cannot be considered separate from the context of people’s lives. The way in which these people express themselves can influence the way they position themselves in society and how they see themselves. When the government in some way omits its role of social protection, it creates situations of oppression in inequality in access to policies and services [43].

For many years, the allocation and distribution of health professionals, especially physicians, have been considered as an important barrier to increasing coverage and access to PHC [11]. The shortage of physicians mainly occurs in areas of greater social vulnerability, which include regions that are difficult to access, remote, and with lower income [12, 13]. A study has recently revealed that 90% of the Brazilian physicians who graduated from municipalities with less than 100 000 inhabitants did not stay in these locations [14]. In other countries, professional migration is justified by the pursuit of better salaries, better living and working conditions, and employment, in addition to earning more experience [15]. For this reason, in recent years, the Brazilian Federal Government has sought to establish strategies for the settlement and allocation of physicians in regions with low medical coverage, such as the “PHC Valorization Program” (in Portuguese, PROVAB) and the “*Mais Médicos* (More Doctors) Program” (in Portuguese PMM, in English “More Doctors Program”) [16].

### The *Mais Médicos* Program and equity

The PMM is considered one of the most relevant initiatives implemented in the country in order to expand and develop the PHCs and to reduce the disparities in health care. The program was inaugurated in 2013; within its lines of actions, one finds the emergency provision of physicians in remote or difficult to access areas and/or populations of greater vulnerability, according to some criteria, among which are municipalities with 20% or more of their population in extreme poverty and areas of high social and economic vulnerability in the capital cities and metropolitan regions (MR) [17, 18].

By the end of 2015, 73% of the Brazilian municipalities [19] had already been taking part in the PMM. The reduction of social and regional inequalities depends on the recognition of the needs of each population group, and it is based on equity, one of the principles of the SUS to guarantee and sustain the right to health care, which is envisaged by the Brazilian public policies [20]. Among the diversity of the proposed concepts, equity can be translated into actions carried out to reach everyone, in an equal and even way, but also in treating the unequal in an appropriate way, prioritizing those who are at more disadvantage owing to previous conditions, especially economic and related to social capital, as well as groups at higher risk [21]. In this respect, it is possible to infer that the offer of services and the availability to access it by the population is also determined by the sociodemographic characteristics of different population groups within their territorial contexts [22].

Some authors refer the PMM was a response to the movement “Where is the Doctor?” launched by the National Front of Mayors in February 2013, in which the mayors claimed about the difficulties in recruiting and maintaining physicians in their remote regions and areas with higher social vulnerability [2, 23, 24]. Other authors state that the Program emerged from a window of opportunity created by the national-level social manifestations that occurred in 2013, which claimed for improvement in the health care in general and in addition to other various demands such as urban mobility, free transportation pass, education, democratization of media, and public security [25].

In this context, the PMM was created and implemented by means of a provisional bill in June 2013, and more than 14 000 physicians were allocated in the first 12 months of the Program, mainly in these regions of greater vulnerability and where it was difficult to attract professionals [2, 26] specially those municipalities with 20% or more of their population living in extreme poverty [27]. However, there is no information about how the distribution of these professionals took place within the very populous municipalities.

Considering that socioeconomic disparities are a reality in the Brazilian state capitals and MRs, even though they might share boundaries, it is important to investigate the distribution of physicians by the PMM at this geographic level using an equity lens. These results can contribute to increase the knowledge on the PMM and to the discussion on how the program was important to reduce the disparities in the access to the medical health care in Brazil.

### The Social Vulnerability Index—IPEA

In order to map the social vulnerability in the greater MRs, the Institute for Applied Economic Research (in Portuguese, IPEA), based on the social vulnerability indexes of the Brazilian Human Development Atlas, proposed the Social Vulnerability Index (in Portuguese, IVS). The IVS translated the access and the insufficiency of resources enabling the identification of the deficiency in the offering of health services and actions [10]; it is composed of three dimensions: urban infrastructure, human capital (health and education), and income and work. It is calculated on the arithmetical mean of the sub-indexes of each of the dimensions, worked out from the demographic census variables presented by the Brazilian Institute of Geography and Statistics. The IVS vulnerability varies from 0 to 1, where the closest values to 1 represent more vulnerable. The current available IVS is based on data from the 2010 Brazilian National Census [10].

In order to characterize the areas with continual territory and socioeconomic similarities, the IPEA took into account the housing development units (in Portuguese, UDH), put together from data from censitary sectors mapped in the National Census. These units enable us to capture the diversity of context and social features that occur within these spaces portraying inequalities closer to the real scenario. Each one of the UDHs has a unique code, and the calculation of the respective IVS is based on the mean of values of specific variables registered by the sectors which compose each UDH [10].

According to the IPEA, improvement in the social conditions of the population, observed in recent years, took place in a heterogeneous way throughout the Brazilian territory showing that public policies implemented in the last decades were not enough to equate the situation of uneven equity [10].

Moser [28] points out that the wellness of a population and its individuals depends not only on their income, but also on suitable housing, basic sanitation, access to PHC, education, and quality public transportation. The concept of vulnerability used in the setting up of the IVS index takes into account all, and any risks are unevenly distributed among individuals, making those who contribute with less materials and/or symbolic assets more vulnerable [10].

Public policies and health programs aim to put into practice health actions and services in compliance with the guidelines and must seek opportunities so that the right to health care be given to all citizens, according to the principles established by the SUS: integrality, universality, decentralization, and equity, among others [29]. For Carrapato et al., the pursuit of health services and actions reinforces the importance of effective measures so as to mitigate the effects of health determinants on populations [30].

The legislation which grounds the PMM states as its goal to reduce the shortage of physicians in the priority regions for the SUS so as to reduce regional inequalities in health care provision [18]. Thus, it seeks to provide medical assistance available in compliance with the demand requested by the municipal managers.

Given such elements, the present study aims to analyze if the distribution of the physicians from the PMM was compliant with the perspective of equity considering the social vulnerability as an important parameter to be taken into consideration in view of the existing disparities among the populations of the greater Brazilian MRs.

## Methods

This is a cross-sectional study with a quantitative approach based on secondary data. A MR with the largest population for each of the Brazilian geographic regions was selected, namely, South Region: Porto Alegre; Southeast Region: São Paulo; Midwest Region: Distrito Federal (DF); North Region: Manaus; and Northeast Region: Recife. For the purpose of this study, Brasilia, which is the National Capital of Brazil and its 21 surrounding municipalities, was considered in the composition of the Distrito Federal MR, according to the Complementary Law n° 94, February 19, 1998 [31]. The population criteria were chosen instead of density because studies indicate that places with a high demographic density are places with greater ease of urban services [44]. Information regarding the Family Health Teams (in Portuguese, “ESF”) was collected from the “Team Implementation Record” provided by the Primary Health Care Department from the Brazilian Ministry of Health [32]. In order to estimate the ESF population coverage, the parameter of 3450 individuals per team was taken into account, in compliance with the guidelines established by the Ministry of Health [32]. Information about the allocation of physicians and geo-referencing of the primary health units (UBS) is public and is available in the National Registry of Health Establishments (CNES) database; it is made available by the SUS Informatics Department (DATASUS). Data about UBS and IVS physicians were composed with the assistance of the QGIS 3.4.5 Madeira program, which is the same program used in the construction of the maps and they correspond to June 2016, approximately 3 years after the implementation of the Program.

According to the IPEA, there are five levels of vulnerability: IVS values that range from 0 to 0.200 and which rank the UDH as “very low” in vulnerability, from 0.201 to 0.300 and which belong to the “low” category, from 0.301 to 0.400 which is “average,” from 0.401 to 0.500 the “high category,” and from 0.501 and above ranked as “very high” in vulnerability [10]. In some of the studied MRs, the poorest areas present values that do not characterize “very high vulnerability” according to the IVS, which is the case of Porto Alegre and São Paulo. So, the quintile analysis was also carried out for each one of the MR so as to guarantee an equal proportion of UDHs in each cluster. Thus, the first and second quintiles correspond to the groups with the lowest values of vulnerability, the third with intermediary values; the fourth and fifth refer to the UDHs with the highest IVS and, therefore, the most vulnerable.

At first, the UBSs were identified through their codes from the analysis MR obtaining their geo-localization data from the addresses registered with the [33–35], as well as with the *Google Earth* software. Then, each UBS was linked to the UDH where it had been located for the connection with their respective IVS that had characterized the social vulnerability of the context for the insertion of the physician from the Program. In this study, the number of physicians allocated in each UBS was not taken into account, since the objective is limited to the presence of at least one physician from the PMM in each of the units.

The study did not involve the collection of information directly from the research participants, and therefore, the approval from the Ethics Committee was not required. The data used is secondary, and publicly available.

## Results

The average IVS for each MR hides the existing internal inequalities as they present values that are too far between the minimum and the maximum (Table 1). The São Paulo MR stands out; it is the one with the largest population, which, despite the number of ESFs comparatively higher in relation to others, it shows the lowest coverage of ESF (34.0%), while Recife MR appears to have the highest coverage (54.4%).

**Table 1:** Description of the characteristic of the Vulnerability Index and the Family Health Strategy in the analyzed metropolitan regions, 2016, Brazil

Metropolitan region (MR)	IVS^a^ average	Min-Max^a^	2016^b^ population	ESF^c^ number of teams	ESF (%)^d^ coverage
Porto Alegre	0.270	0.065–0.445^e^	4 276 475	602	48.5
São Paulo	0.299	0.040–0.475^e^	21 242 939	2.093	34.0
Distrito Federal	0.322	0.055–0.504	4 284 676	480	38.6
Manaus	0.415	0.083–0.683	2 568 817	315	42.3
Recife	0.392	0.076–0.704	4 019 396	634	54.4

^a^Index of Social Vulnerability calculated by the IPEA

^b^Population estimate for 2016—IBGE

^c^ESF estimate from the Primary Health Care Department (DAB)—Ministry of Health

^d^Coverage based on an average of 3 450 people who usualy have a follow-up by each ESF

^e^In these MRs, there are no areas with IVS > 0.501, that is, with very high vulnerability

Of the 2592 existing UBSs in the five MRs of the study, Table 2 presents striking differences between these regions: in Porto Alegre MR, 73% of the UBSs are located in areas of “low” or “very low” vulnerability. On the other hand, in the Manaus and Recife MRs, the situation is the opposite: the UBSs are founded in areas of “high” and “very high” vulnerability: 58.4% and 57.1%, respectively. The fixed cut-off points were used to compare the five MRs regarding the IVS category; clearly they present different levels of vulnerability among themselves.

**Table 2:** Distribution and percentage of the primary health care units according to the classification by the Index of Social Vulnerability by categories and quintiles in the five metropolitan regions, 2016, Brazil

Distribution of the primary health care units by category according to the Index of Social Vulnerability (*N* and %)						
IVS Category^a^	Porto Alegre^b^	São Paulo^b^	Distrito Federal	Manaus	Recife	Total
Very low	120 (25.5)	117 (12.3)	26 (6.9)	2 (0.7)	22 (4.5)	287 (11.1)
Low	225 (47.9)	295 (30.9)	90 (23.8)	34 (11.4)	34 (6.9)	678 (26.2)
Medium	113 (24.0)	492 (51.6)	166 (43.9)	88 (29.5)	155 (31.5)	1014 (39.1)
High	12 (2.6)	50 (5.2)	90 (23.8)	115 (38.6)	191 (38.8)	458 (17.7)
Very high	0 (0)	0 (0)	6 (1.6)	59 (19.8)	90 (18.3)	155 (6.0)
Total	470 (100)	954 (100)	378 (100)	298 (100)	492 (100)	2.592 (100)
Distribution of the primary health care units by quintiles according to the Social Vulnerability Index (*N* and %)						
Quintiles^c^	Porto Alegre	São Paulo	Distrito Federal	Manaus	Recife	Total
1st	98 (20.9)	175 (18.3)	36 (9.5)	10 (3.4)	29 (5.9)	348 (13.4)
2nd	95 (20.2)	205 (21.5)	71 (18.8)	55 (18.5)	67 (13.6)	493 (19.0)
3rd	130 (27.7)	241 (25.3)	76 (20.1)	71 (23.8)	99 (20.1)	617 (23.8)
4th	88 (18.7)	215 (22.5)	73 (19.3)	71 (23.8)	131 (26.6)	578 (22.3)
5th	59 (12.6)	118 (12.4)	122 (32.3)	91 (30.5)	166 (33.7)	556 (21.5)
Total	470 (100)	954 (100)	378 (100)	298 (100)	492 (100)	2.592 (100)

^a^IPEA’s IVS categories: very high > 0.500, high 0.500 to 0.401, medium 0.400 to 0301, low 0.300 to 0.201, and very low < 0.200

^b^Nonexistent areas with very high vulnerability in the Porto Alegre and São Paulo metropolitan regions

^c^IVS quintiles: Porto Alegre 1st 0.065 to 0.180, 2nd 0.190 to 0.230, 3rd 0.240 to 0.290, 4th 0.300 to 0.340, and 5th 0.350 to 0.445; São Paulo 1st 0.055 to 0.220, 2nd 0.230 to 0.290, 3rd 0.300 to 0.350, 4th 0.360 to 0.390, and 5th 0.400 to 0.504; Distrito Federal 1st 0.040 to 0.230, 2nd 0.240 to 0.290, 3rd 0.300 to 0.340, 4th 0.350 to 0.380, and 5th 0.390 to 0.475; Manaus 1st 0.083 to 0.250, 2nd 0.260 to 0.330, 3rd 0.340 to 0.410, 4th 0.420 to 0.470, and 5th 0.480 to 0.686; and Recife 1st 0.076 to 0.260, 2nd 0.270 to 0.320, 3rd 0.330 to 0.390, 4th 0.400 to 0.460, and 5th 0.470 to 0.704

In the data organized by quintiles, the analysis is carried out within the reality of each MR, where the highest vulnerability is observed in the 4th/5th quintiles and the lowest vulnerability in the 1st/2nd quintiles.

Comparing with the analysis by category, it seems that standardized categorization can hide existing disparities in the distribution of units in different areas within each MR; in this case, the values “average” for the category stand out. According to the IVS categorization, the Porto Alegre and São Paulo MRs do not have areas of “very high” vulnerability.

In Manaus, 76.2% of the physicians worked in the “high” or “very high” vulnerability unit; on the other hand, 4.8% of the physicians from the PMM were allocated in units located in “low” vulnerability areas and none of them at “very low” vulnerability sites (Table 3). The São Paulo MR was the one which hosted more physicians (369), of which, 62.9% were allocated in regions with “medium” vulnerability. In Recife, 63.9% of the physicians worked in a unit of “high” or “very high” vulnerability.

**Table 3:** Percentage of primary health units with physicians from the Program allocated according to the classification of the Social Vulnerability Index by category and quintile, at the five metropolitan regions, 2016, Brazil

Primary health unit percentage with physicians from the PMM by the Index of Social Vulnerability by category						
IVS^a^ category	Porto Alegre^b^	São Paulo^b^	Distrito Federal	Manaus^c^	Recife	Total
Very low and low vulnerability [IC_95%_]	65.8^**e**^ [59.3–72.4]	29.5 [23.3–35.8]	26.8 [20.8–32.9]	4.8 [1.9–7.6]	4.1 [1.4–6.7]	30.6 [24.5–36.7]
Medium vulnerability [IC_95%_]	29.7 [23.4–36.0]	62.9^**e**^ [56.2–69.5]	45.3^**e**^ [38.5–52.0]	19.0 [13.8–24.3]	32.0 [25.8–38.2]	44.4 [37.9–51.0]
High and very high vulnerability [IC_95%_]	4.5 [1.6–7.3]	7.6 [4.0–11.2 ]	27.9 [21.9–34.0]	76.2^**e**^ [70.5–81.9]	63.9^**e**^ [57.6–70.3]	25.0 [19.3–30.7]
Total % (*n*)	100% (202)	100% (369)	100% (179)	100% (84)	100% (147)	100% (981)
Primary health unit percentage with physicians from the PMM by the Index of Social Vulnerability by quintile						
IVS^d^ quintiles	Porto Alegre	São Paulo	Distrito Federal	Manaus	Recife	Total
1st and 2nd [IC_95%_]	31.7 [25.3–38.1]	25.5 [21.0–29.9]	24.6 [18.3–30.9]	9.5 [3.2–15.8]	9.5 [4.8–14.3]	22.8 [20.2–25.5]
3rd [IC_95%_]	29.2 [22.9–35.5]	28.7 [24.1–33.3]	22.9 [16.7–29.1]	19.0 [10.7–27.4]	19.0 [12.7–25.4]	25.5 [22.8–28.2]
4th and 5th [IC_95%_]	39.1 [32.4–45.8]	45.8^**e**^ [40.7–50.9]	52.5^**e**^ [45.2–59.8]	71.4^**e**^ [61.8–81.1]	71.4^**e**^ [64.1–78.7]	51.7 [48.6–54.8]
Total % (*n*)	100% (202)	100% (369)	100% (179)	100% (84)	100% (147)	100% (981)

^a^IPEA’s IVS categories: very high > 0.500, high 0.500 to 0.401, medium 0.400 to 0301, low 0.300 to 0.201, and very low < 0.200

^b^Nonexistent areas with very high vulnerability in the Porto Alegre and São Paulo metropolitan regions

^c^Nonexistent physicians from the PMM allocated in the UBSs at areas of very low vulnerability in the Manaus metropolitan region

^d^IVS quintiles: Porto Alegre 1st 0.065 to 0.180, 2nd 0.190 to 0.230, 3rd 0.240 to 0.290, 4th0.300 to 0.340, and 5th 0.350 to 0.445; São Paulo 1st 0.055 to 0.220, 2nd 0.230 to 0.290, 3rd 0.300 to 0.350, 4th 0.360 to 0.390, and 5th 0.400 to 0.504; Distrito Federal 1st 0.040 to 0.230, 2nd 0.240 to 0.290, 3rd 0.300 to 0.340, 4th 0.350 to 0.380, and 5th 0.390 to 0.475; Manaus 1st 0.083 to 0.250, 2nd 0.260 to 0.330, 3rd 0.340 to 0.410, 4th 0.420 to 0.470, and 5th 0.480 to 0.686; Recife 1st 0.076 to 0.260, 2nd 0.270 to 0.320, 3rd 0.330 to 0.390, 4th 0.400 to 0.460, and 5th 0.470 to 0.704

^e^IC_95%_ shows that there is a statistically significant difference between this proportion and further proportions in the column

When analyzing the 95% confidence intervals calculated for each IVS distribution category (Table 3), it is observed that there is a statistically significant difference in the MR in Porto Alegre, indicating a higher proportion of PMM physicians allocated in UBSs in regions with “low” or “very low” vulnerability (totaling 65.8%). On the other hand, in the MRs of São Paulo and DF, there was a significantly higher number of physicians from the PMM (62.6% and 45.3%, respectively) in the “medium” vulnerability UBSs. In the MRs of Manaus and Recife, 76.5% and 63.9% of the proportion of PMM physicians (significant) were allocated to areas of “high” or “very high” vulnerability, respectively. According to these criteria, only the last two RMs prioritized the most vulnerable areas.

Analyzing the distribution of UBSs with physicians from the PMM allocated by quintiles of vulnerability and intervals of confidence (IC_95%_) calculated for each MR, it is noticed that the Recife, Manaus, and the DF MRs, the 4th and 5th quintiles (the most vulnerable), hosted physicians in a significantly higher proportion than the other quintiles, namely 71.4%, 71.4%, and 52.5%, respectively, all of which exceed the national average (51.7%). It is worth mentioning that in the São Paulo MR, the UBSs located in the high vulnerability quintiles (4th and 5th) also hosted physicians in a significantly higher proportion than others (45.8%); despite being significant, this proportion did not reach 50%, that is, in this RM, most UBSs with physicians are located in the areas of the smallest quintiles (1st, 2nd, and 3rd, which together represent 54.2%). There was no significant difference in the allocation of physicians in the MR in Porto Alegre, indicating that there was no prioritization of UBSs according to the vulnerability.

With the exception of the South Region MR, historically regarded as the most developed region in terms of economy, the other MRs, São Paulo, the DF, Manaus, and Recife, showed a highly proportionate distribution of physicians in areas of high vulnerability. We highlight Recife as the place with the greater quantity of physicians allocated in units with the highest IVSs from the sample.

**Fig. 1:**
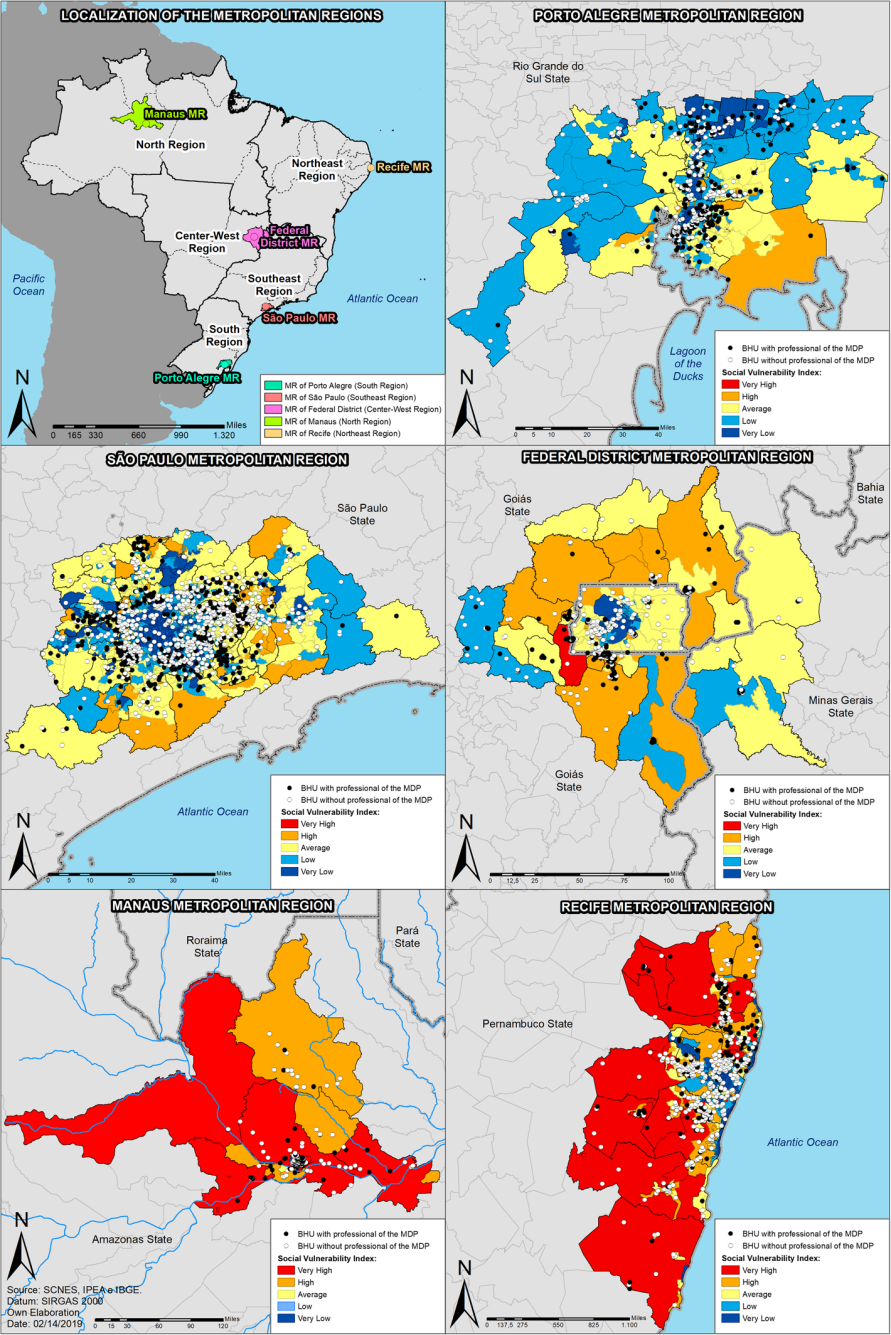
Localization of the Mais Medicos Doctors in the metropolitan regions and the primary health units in the Housing Development Units, according to the IVS categories, 2016, Brazil

**Fig. 2:**
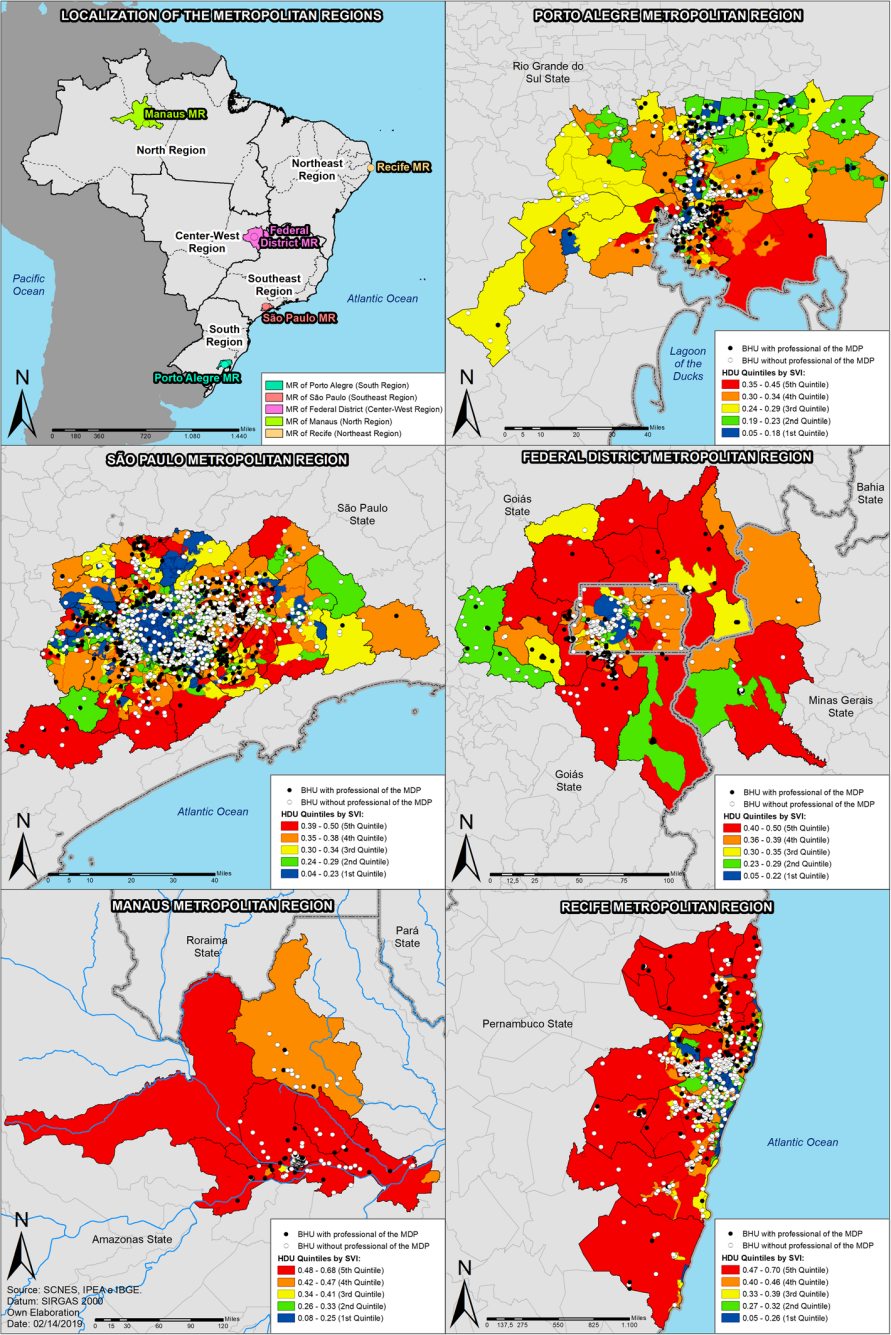
Localization of the Mais Médicos Doctors in the metropolitan regions and the primary health units in the Housing Development Units, according to quintile, 2016, Brazil

According to the findings, it is possible to suggest that the Porto Alegre MR prioritized the allocation of physicians in areas of “very low” and “low” vulnerability, that is, areas which were not a priority for the PMM. Still within this context, São Paulo, the Distrito Federal, Manaus, and Recife allocated physicians in areas of “high” and “very high” IVS. This trend can also be seen in the maps categorized by quintiles, which enables the visualization and localization of these areas, usually, peripheral. Figures 1 and 2 (available in a larger size in the supplementary material 1) make it evident how much the same region, even with a large population and/or strong economy, can present socioeconomic inequalities and consequently have groups of citizens who live under extreme indexes of vulnerability which impact on the limitation to access to the health care services. From the maps, Manaus and Recife MRs show the largest area of their territories affected by high vulnerability, according to the red and orange color displayed, in addition to the UDH concentration with the best values for the central regions of each MR.

The use of the quintiles was justified by observing the different realities portrayed in the MRs: the most vulnerable areas in Porto Alegre (0.445) differ in relation to the highest indexes in Recife (0.704) and in relation to the category previously determined by the IVS from IPEA. Nevertheless, even though the indexes regarded as the highest in Porto Alegre falls in the category “high” and not “very high” vulnerability, this is the most critical area observed within this region and should be prioritized, as established by the PMM’s normatives.

## Discussion

Based on the availability of sociodemographic information and health data, it is possible to carry out studies to investigate how the health care processes affect a population, in addition to discussing the results of the initiatives to expand medical assistance. In this work, specifically, it was possible to highlight that the PMM initiative contributed to the availability of PHC in regions with different degrees of social vulnerability, focusing on the most vulnerable areas. On the other hand, the findings showed the absence of UBSs in areas with a higher Index of Social Vulnerability in the largest MRs (Sao Paulo and Porto Alegre).

The importance of a more cautious attention becomes clearer when analyzing whether the distribution of physicians of the PMM within the MRs met the priority criteria for the poor and vulnerable areas set in the official documents for the program, according to the criteria of equity. This is so because extreme values of vulnerability tend to create average values that hide important realities that, at times, may go unnoticed in the setting up of strategies to reduce inequity, which are most often created at municipal levels. According to the findings presented by this study, in three MRs, Manaus, Recife, and the DF, most of the UBSs were located in peripheral and most vulnerable areas. It was also found that the allocation of physicians occurred mainly in these regions, in line with the Program’s normative framework and with other studies’ which have already pointed out that municipalities with extreme poverty—that is, highly vulnerable—were to be prioritized in the implementation of the PMM so that it would assist the populations with greater needs of attention [36].

The results of this study in addition to further research suggest that conditions were provided for the PMM to produce positive results regarding the expansion of coverage and the reduction of inequality to access the system, especially in regions with shortage of these professionals, mainly in the North and Northeast regions [37], besides the potential contribution for a more geographic equitable distribution to address resources, considering the most vulnerable areas, also considering the improvement in the infrastructure of the UBS [38]. The PMM was even more important to supply the needs regarding the lack of medical professionals in various regions throughout Brazil, with special emphasis in the most vulnerable areas [39], as it had been determined by its priority goals, once the highest concentration of participating physicians was found in the municipalities of extreme poverty: a number threefold higher than in the capital cities and richest municipalities [28].

Studies have pointed out the significant evolution of the ESF and the PMM [40, 41], and have indicated that the increase in the number of physicians in the ESF enabled a greater effectiveness and more equity in the primary level of care attention, considering the hospitalizations regarding outpatient care, especially for more socially vulnerable populations and/or where there is a lack of these professionals [27]. More than 50% of the implemented ESF in Brazil in 2012 were incorporated into the PMM, and most of these teams were from municipalities with less than 30 000 inhabitants, providing the chance to improve access to health for these populations.

The establishment of the PMM increased the coverage of health care in about 100% of the population in small municipalities resulting in the decrease in referrals to specialists and urgent care and a reduction in the waiting time between the request of an appointment and the day of the appointment [2, 42]. These results corroborate the results of this analysis; they confirm the prioritization in areas of greater resource inequities, in the access and health care, not only in the municipal level, but sub-municipal too.

Despite the fact that results from previous studies have shown municipalities where extreme poverty was greater had been prioritized in the implementation of the PMM, this is the first study that analyzed the sub-municipal level to consider a social vulnerability index as a parameter to characterize aspects of equity in the distribution of the Program’s physicians. The results presented by this study show that in the MR analyzed, despite the presence of the immense socioeconomic inequalities, the PMM aimed to prioritize those areas that were more unprivileged, therefore contributing to the reduction of disparities in the access to PHC. The health policies within a universal system such as the Brazilian SUS may work as a redistributing mechanism, in as much as it reduces disparities of access and, consequently, it favors equity in the country’s health care. Thus, as stated by Giovanella and her collaborators, it is necessary to study and to get to know the health systems, as well as their programs and strategic performance as it allows us to know how and where their structure fail or success in achieving the improvement of the health conditions of the populations [29]. The coverage area and action of each ESF can also be taken into account, as well as the number of physicians allocated for each MR to justify, for instance, the non-significant prioritization of physicians in more vulnerable areas of large cities, such as this is the case of the Porto Alegre MR.

Limitations of the present study include the fact that the data used for the IVS calculus, despite being the most recent currently available, correspond to the 2010 Census, and the base year for the study is 2016. It must be taken into account that some factors may have been time-influenced, influenced by the demographic transition among other features that might have altered the vulnerability profile of the population along these 6 years. Another limitation might be a reflection of some inaccuracies of the UBS geographic location of data and the distribution of physicians available in the official databases. At last, for this study, the number of the PMM’s physicians allocated in each UBS was not taken into consideration, but only the presence of the PMM in the UBSs.

## Conclusions

New studies should be carried out so as to further research the issues raised by the present study, especially aiming to provide more evidence of the effects of the PMM, which would enable a better assessment of the effectiveness of the Program and the APS in Brazil. In this context, it is worth highlighting that the equity in the results, besides its use as an informing principle for the elaboration of policies, is an aspect of high relevance to present the effects of the Program for the welfare assistance and in the reduction of inequities in the access; it also serves as a subsidy for the discussion of policies of provision and retention of professionals, especially in the least assisted, remote, and vulnerable areas.

It can be accredited to the PMM, in addition to its other objectives such as medical training and the structuring of a humanized curriculum, that it enabled people from remote and difficult-to-reach regions and citizens from the outskirts of the MRs as they had improved their access to medical services. It deeply mattered in the upgrade of life quality for the most vulnerable population, even for those citizens who live in the capital cities. The PMM was probably not able to solve all health care access issues and barriers, but it has enabled socially excluded people to count on the assistance of the Program’s physicians. It is worth mentioning that the equity in the attention to health care depends on a process that involves the integration of other levels of care, it must take into account issues that go beyond social problems triggered in the populations, the problems the Brazilian health system itself is facing, whether structural, institutional, or political. Knowing the pattern of distribution of a population facilitates the decision-making in the elaboration of policies and further actions and services offered equally, prioritizing the more unprivileged citizens and those who are at risk.

## Supplementary information

**Additional file 1:.** Larger Figure S1.

**Additional file 2:.** Larger Figure S2.

## Data Availability

The data that support the findings of this study are available on request from the authors.

## Abbreviations

CNES: National Registry of Healthcare Establishments
DATASUS: Department of Information Technology of SUS
DF: Federal District
ESF: Family Health Teams
IBGE: Instituto Brasileiro de Geografia e Estatística
IPEA: Institute for Applied Economic Research
IVS: Social Vulnerability Index
MRs: Metropolitan regions
PHC: Primary health care
PMM: *Mais Médicos* Program (More Doctors Program)
PROVAB: Primary Health Care Valorization Program
SUS: Unified Health System
UBS: Primary health care units
UDH: Housing development units
