# Supplementary figures and images for: Spatial distribution of the “Mais Médicos (More Doctors) Program” and social vulnerability: an analysis of the Brazilian metropolitan regions
Source: Hum Resour Health. 2020 Aug 5;18:57. doi: 10.1186/s12960-020-00497-5 (PMC7409470; doi:10.1186/s12960-020-00497-5)

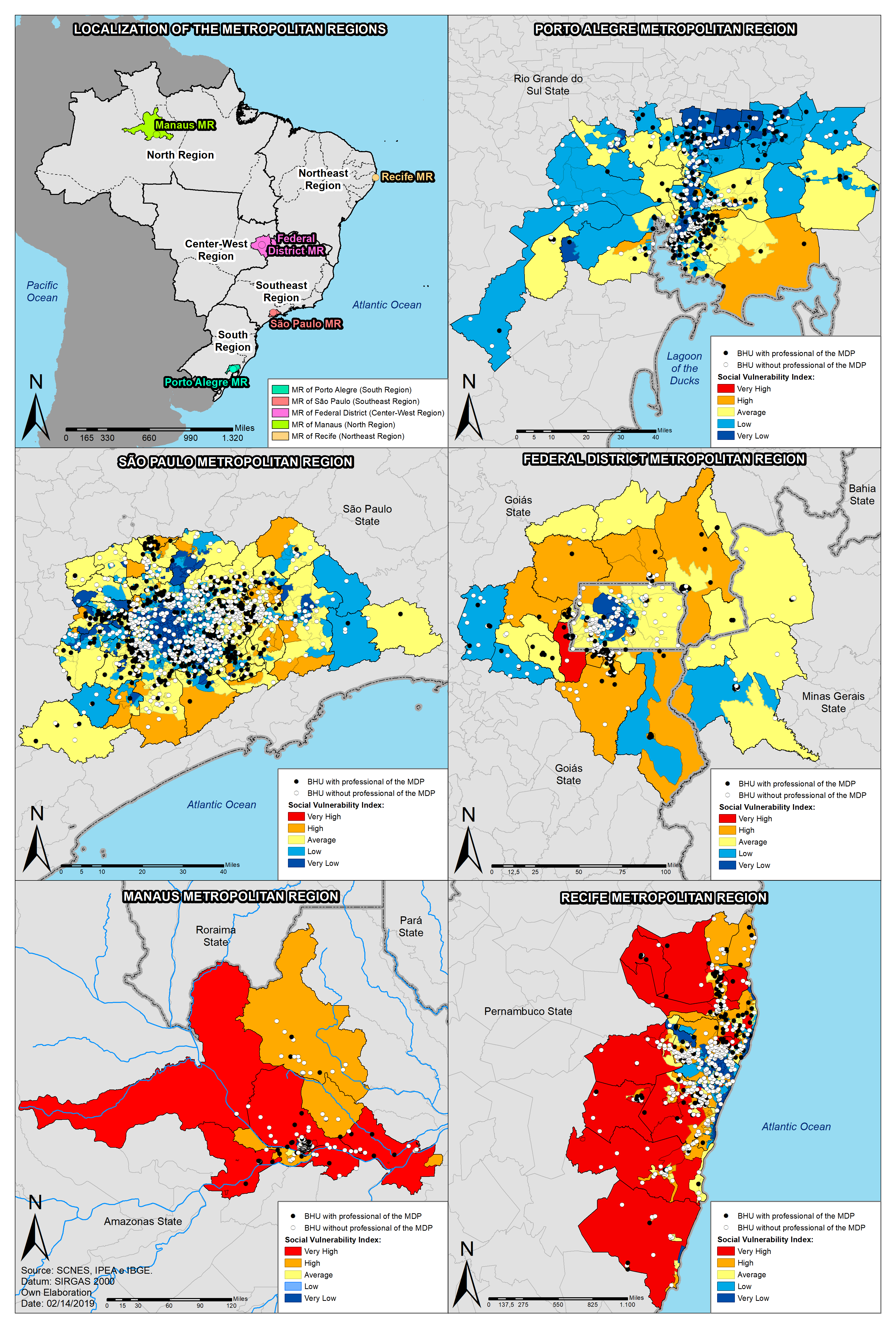

Supplement: Supplementary file 1 — Additional file 1:. Larger Figure S1. [file 12960_2020_497_MOESM1_ESM.png]

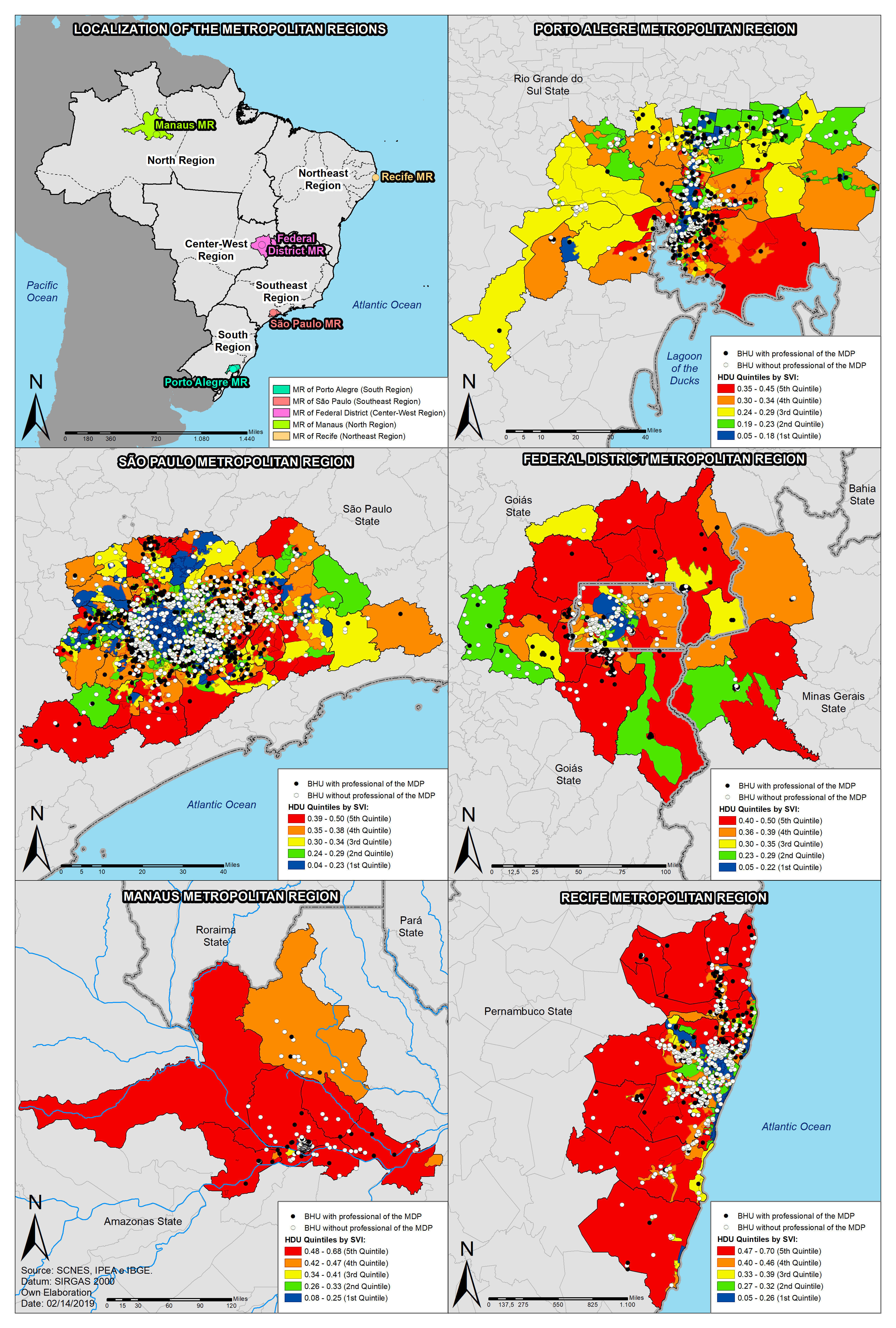

Supplement: Supplementary file 2 — Additional file 2:. Larger Figure S2. [file 12960_2020_497_MOESM2_ESM.png]
